# Retrospective review: single- and multidonor washed microbiota transplantation have equivalent efficacy in the treatment of autism

**DOI:** 10.3389/fcimb.2025.1606417

**Published:** 2025-07-10

**Authors:** Ya-Mei Zheng, Meng-Meng Ye, Hong-Ying Zhang, Dan-Ping Luo, Tao Liu, Xing-Xiang He, Xian-Yun Chen, Li-Hao Wu

**Affiliations:** ^1^ Department of Gastroenterology, The First Affiliated Hospital of Guangdong Pharmaceutical University, Research Center for Engineering Techniques of Microbiota-Targeted Therapies of Guangdong Province, Guangdong, Guangzhou, China; ^2^ Department of Gastroenterology, The First People's Hospital of Shaoguan, Shaoguan, Guangdong, China

**Keywords:** washed microbiota transplantation, autism, short-term efficacy, faecal flora, single donor, multidonor

## Abstract

**Background:**

Autism spectrum disorder (ASD) is a serious neurodevelopmental disorder with no effective treatment. This study explored the short-term clinical effects of washed microbiota transplantation (WMT) with different numbers of donors on autism.

**Methods:**

Consecutive ASD patients treated with two continuous WMT courses from March 2020 to March 2022 at the First Affiliated Hospital of Guangdong Pharmaceutical University were retrospectively assessed. Basic information, aberrant behavior checklist (ABC) scores, childhood autism rating scale (CARS) scores, sleep disturbance scale for children (SDSC) scores, adverse reactions, and feces were collected.

**Results:**

Forty-four patients were included (single-donor group: 17 patients; multidonor group: 27 patients). The CARS, ABC and SDSC scores didn’t differ between the two groups before treatment. After two courses, the scores for the 44 patients were lower than those at baseline (P<0.05), with no severe adverse reactions observed. After the first course, the mean ABC (P=0.049) and SDSC (P=0.019) scores were significantly different between the single-donor and multidonor groups, but the difference disappeared after two courses. The alpha-diversity of the faecal flora in the effective-group was greater than that in the ineffective-group (Shannon index P=0.0018). *Lactobacillus* was the predominant genus in the effective group, whereas *Faecalibacterium, Campylobacter*, and *Sphingomonas* were predominant genera in the ineffective group.

**Conclusion:**

After two WMT courses, the symptoms of ASD improved, with good short-term treatment efficacy. The ASD symptom improvement did not differ between the single-donor and multidonor groups. Changes in the alpha-diversity and abundance of the faecal microbiota after WMT may be related to treatment efficacy.

## 1 Introduction

Autism spectrum disorder (ASD) is a severe neuropsychiatric developmental disorder characterized by a narrow range of interests or stereotyped patterns of behavior and problematic communication and language use in social interactions (Sanlier and Kocabas, 2023). Epidemiological studies have shown that the incidence of ASD is increasing annually (Wang and Fu, 2023). For children with ASD with strong plasticity, early recognition and intervention with standard treatment can improve involvement in social life; thus, early diagnosis and early treatment are highly important. At present, the aetiology of autism is not clear, and it may be related to genetic factors, immune regulation disorders, inflammation and environmental toxin exposure (Han et al., 2021).

The available research and literature on the diagnosis and treatment of autism lack a significant effective therapeutic method for this condition. Relatively effective methods for the treatment of autism include nondrug therapy (behavior, speech and social therapy), drug therapy and diet therapy. Drug therapy for ASD includes the use of antipsychotics, antidepressants, mood modulators, and stimulants (Farmer et al., 2013), which are used mainly to control secondary symptoms associated with ASD, but no drug has been approved to treat the core symptoms of ASD. Nondrug therapy for ASD includes educational, behavioral or communication-based strategies, which are used alone or in combination as part of an individualized plan to improve learning and community engagement. Various ASD symptom classifications have been developed to select and employ different nonpharmacological therapies, but no consensus has been reached to date.

Pharmacological treatment of ASD has substantial side effects and lacks satisfactory efficacy in the treatment of the core ASD symptoms. Nonpharmacological treatment requires strong cooperation from families and communities, and clinical data supporting its effectiveness is lacking.

The reciprocal interactions among the central nervous system, enteric nervous system and gastrointestinal tract are collectively called the gut–brain axis (Gao et al., 2020). Recent research suggests that gut microbes are involved in the dual-directional regulation of the gut–brain axis and central nervous system through nerve, endocrine, metabolic, and immune pathways, which suggests that microecological therapy modifies the intestinal flora to affect the gut–brain axis and relieve symptoms of ASD in patients (Osadchiy et al., 2019).

Potential therapies for autism that regulate the intestinal flora include probiotics and prebiotics, microbiota transfer therapy, and faecal microbiota transplantation (FMT), as well as diet modifications and antibiotic use (Sanlier and Kocabas, 2023). Clinical studies have shown that treatments that modulate the gut microbiota can improve ASD symptoms. At present, the factors contributing to the enhanced therapeutic effect of washed microbiota transplantation (WMT) for autism depend mainly on the duration of treatment and concurrent gastrointestinal symptoms (Pan et al., 2022; Hu et al., 2025). This study explored the relationship between WMT treatment for autistic patients and their donors.

## 2 Materials and methods

### 2.1 Participants

Patients with ASD who received at least 2 courses of WMT at the First Affiliated Hospital of Guangdong Pharmaceutical University from March 2020 to March 2022 were retrospectively enrolled. For this study, informed consent was obtained from guardians, and the study was approved by the First Affiliated Hospital of Guangdong Pharmaceutical University: Medical Ethics Review [2020] No. 68.

The inclusion criteria were as follows: patients aged 2–18 years, male or female sex, guardian consent to be obtained at follow-up, patients diagnosed with ASD according to the 5th edition of the Diagnostic and Statistical Manual of Mental Disorders, and patients with complete clinical data.

The exclusion criteria were as follows: patients with diseases such as Rett syndrome, infantile dementia, Tourette syndrome, selective mutism, and childhood depression; patients with ASD combined with schizophrenia, bipolar disorder and other major mental diseases; and patients with serious heart and kidney diseases, autoimmune diseases or other diseases that seriously affect quality of life.

### 2.2 Methods

All patients received 6 consecutive days of WMT through transendoscopic enteral tubing (TET) for each course after completing endoscopic intestinal TET catheterization, followed by a second course of WMT 4 weeks later, with the same method. In this study, 17 healthy people who passed the screening were used as donors. The single-donor group was a group of patients for whom washed microbiota suspensions provided by only one donor were used for the two courses of WMT treatment, whereas patients in the multidonor group received washed microbiota suspensions from two or more donors in the WMT treatment. The method for preparing the bacterial mixture was consistent with the Nanjing Washed Faecal Microbiota Transplantation Methodology Consensus (Nanjing consensus on methodology of washed microbiota transplantation, 2020). All transplantations were performed via a TET in the lower digestive tract. Intestinal preparation for the children was performed as described in “Intestinal Preparation for Digestive Endoscopy in Chinese Children” (Enqiang, 2021).

### 2.3 Data collection

The legal guardians of the enrolled patients signed an informed consent form before the patients underwent WMT. General information on the enrolled patients, including sex and age, was collected. Clinical symptoms of ASD were assessed before and after WMT via questionnaires, including the ABC, CARS, and SDSC, which assess ASD symptoms, such as social behaviors, emotional disorders, and sleep efficacy. Stool samples were collected from 35 patients before and after WMT, and all samples were stored at -80°C after collection and subsequently subjected to 16S RNA sequencing for abundance, alpha-diversity, beta-diversity, and other analyses.

### 2.4 Statistical analysis

The statistical analysis software SPSS 22.0 was used to analyze the data. The mean ± standard deviation (x ± s) was used for normally distributed data, and the median, interquartile range and percentage were used for nonnormally distributed data. Measurement data that met the normality and homogeneity of variance criteria simultaneously were analyzed by independent sample t tests and paired sample t tests; otherwise, the Wilcoxon test was used. Pearson’s test was used for correlation analysis. P < 0.05 was regarded as indicative of statistical significance. The results of population species annotation were visualized with GraPhlAn software.

## 3 Results

A total of 44 ASD patients who met the diagnostic criteria received two courses of WMT, including 17 patients in the single-donor treatment group and 27 patients in the multidonor treatment group. A comparison of the baseline data of the single-donor and multidonor groups revealed that the mean age (6.47 ± 3.777 vs. 6.11 ± 3.142), mean ABC score before WMT (60.17 ± 21.31 vs. 63.81 ± 22.70), CARS score before WMT (36.09 ± 3.49 vs. 35.96 ± 5.37) and SDSC score before WMT (50.07 ± 12.18 vs. 52.19 ± 15.56) were not significantly different (P > 0.05) (**Table 1**).

**Table 1:** Comparison of baseline data between the two groups of patients before WMT treatment.

Item	Single-donor group	Multidonor group	Z	P
Male: Female	16:1	22:5		
Age (year)	6.47 ± 3.777	6.11 ± 3.142	T=0.342	0.734
Pretreatment ABC score	60.18 ± 21.31	63.81 ± 22.70	-0.53	0.599
Pretreatment CARS score	36.09± 3.49	35.96 ± 5.37	0.085	0.932
Pretreatment SDSC score	50.70± 12.18	52.19± 15.56	-0.333	0.741

### 3.1 Overall efficacy of WMT in ASD patients

After one or two courses of WMT, the ABC scores for the 44 patients decreased (baseline vs. 1st course vs. 2nd course: 62.41 ± 22.00 vs. 53.73 ± 20.43 vs. 52.18 ± 19.61, P<0.01) compared with those at baseline before treatment, indicating that ASD symptoms were alleviated after the first course of WMT in ASD patients. Among the 5 aspects of the ABC, the improvements in sensitivity, motor ability and self-care skills were more pronounced, with p values of 0.013, 0.025, and < 0.001, respectively. Compared with those after the first course of treatment, the ABC scores after 2 treatment courses (52.18 ± 19.61 vs. 53.73 ± 20.43) were slightly different (P > 0.05), indicating that as the number of treatment courses increased, the ABC scores decreased (**Table 2**).

**Table 2:** Comparison of the ABC scores before and after WMT.

Item		ABC scores	t	P
Baseline vs. 1st course		62.41 ± 22.00 vs. 53.73 ± 20.43	3.847	<0.001
Baseline vs. 2nd course		62.41 ± 22.00 vs. 52.18 ± 19.61	4.53	<0.001
1st course vs. 2nd course		53.73 ± 20.43 vs. 52.18 ± 19.61	0.63	0.53
Baseline vs. 1st course	sensibility	10.11 ± 5.12 vs. 8.36 ± 5.16	2.586	0.013
	motor ability	11.61 ± 8.13 vs. 9.64 ± 6.75	2.326	0.025
	self-care skill	12.09 ± 4.78 vs. 9.54 ± 4.44	4.046	<0.001
Baseline vs. 2nd course	associates ability	13.64 ± 6.87 vs. 11.66 ± 6.91	2.09	0.042
	motor ability	11.61 ± 8.13 vs. 7.80 ± 6.69	4.03	<0.001
	self-care skill	12.09 ± 4.78 vs. 9.91 ± 5.56	2.39	0.022

After one or two courses of WMT, the CARS scores for the 44 patients decreased (baseline vs. 1st course vs. 2nd course: 36.01 ± 4.69 vs. 33.90 ± 4.31 vs. 33.80 ± 4.23, P<0.01) compared with those before treatment, indicating that the overall clinical symptoms were much improved after the first course of WMT in ASD patients. Interpersonal relationships, imitation, physical application ability, relationships with inanimate objects, adaptation to environmental change, language communication, and general impressions were markedly improved (P <0.05). Compared with those after the first course of treatment, the CARS scores after the second course (33.80 ± 4.23 vs. 33.90 ± 4.31, P > 0.05) decreased slightly, indicating that as the number of treatment courses increased, the CARS scores tended to decrease (**Table 3**).

**Table 3:** Comparison of the CARS scores before and after WMT.

Item		CARS scores	t	P
Baseline vs. 1st course		36.01 ± 4.69 vs. 33.90 ± 4.31	5.02	<0.001
Baseline vs. 2nd course		36.01 ± 4.69 vs. 33.80 ± 4.23	5.33	<0.001
1st course vs. 2nd course		33.90 ± 4.31 vs. 33.80 ± 4.23	0.24	0.81
Baseline vs. 1st course	Interpersonal relationship	2.61 ± 0.56 vs. 2.40 ± 0.52	3.18	0.003
	Imitation	2.40 ± 0.51 vs. 2.25 ± 0.51	2.17	0.036
	Physical application ability	2.51 ± 0.57 vs. 2.31 ± 0.60	2.45	0.018
	Relationship with inanimate objects	2.50 ± 0.55 vs. 2.28 ± 0.50	2.59	0.013
	Adaptation to environmental change	2.18 ± 0.52 vs. 2.00 ± 0.39	2.5	0.017
	Language communication	2.92 ± 0.63 vs. 2.69 ± 0.69	2.03	0.049
	General impression	2.39 ± 0.48 vs. 2.24 ± 0.44	2.23	0.031
Baseline vs. 2nd course	Relationship to nonliving objects	2.50 ± 0.55 vs. 2.30 ± 0.58	2.94	0.005
	Adaptation to environmental change	2.18 ± 0.52 vs. 1.89 ± 0.54	3.1	0.003
	Sense of proximity	2.27 ± 0.70 vs. 2.09 ± 0.57	2.07	0.044
	Nonverbal communication	2.25 ± 0.68 vs. 2.07 ± 0.58	2.11	0.041

After one or two courses of WMT, the SDSC scores for the 44 patients decreased (baseline vs. 1st course vs. 2nd course: 51.61 ± 14.22 vs. 48.27 ± 14.18 vs. 46.11 ± 12.59, P<0.05) compared with those before treatment, which indicated that sleep continuously improved after the two courses of WMT in ASD patients. Trouble falling asleep and sleep disorders were dramatically improved (baseline vs. 1st course: 18.14 ± 5.78 vs. 15.68 ± 4.57, P=0.001). The mean SDSC score of the 2nd course was lower than that of the 1st course, suggesting that the sleep of the ASD patients improved consistently (**Table 4**).

**Table 4:** Comparison of the CARS scores before and after WMT.

Item	SDSC scores	t	P
Baseline vs. 1st course	51.61 ± 14.22 vs. 48.27 ± 14.18	2.22	0.031
Trouble falling asleep and sleep disorders	18.14 ± 5.78 vs.15.68 ± 4.57	3.49	0.001
Baseline vs. 2nd course	51.61 ± 14.22 vs. 46.11 ± 12.59	3.3	0.002
1st course vs. 2nd course	48.27 ± 14.18 vs. 46.11 ± 12.59	1.23	0.23

### 3.2 Comparing the efficacy of single- or multidonor transplantation

After the first WMT, the single-donor group had significantly lower mean ABC scores and SDSC scores than did the multidonor group (ABC score: 53.29 ± 16.90 vs. 54 ± 22.69, P=0.049; SDSC score: 47.76 ± 10.24 vs. 48.59 ± 16.35, P=0.019). However, the difference in the ABC score and SDSC score between the two groups disappeared after the 2nd WMT course. The CARS scores did not significantly differ after one or two WMT courses in either group (**Table 5**).

**Table 5:** ABC, CARS, SDSC scores comparison of single and multiple donor group after WMT treatment.

Item	Single-donor group (n=17) vs. multidonor group (n=27), 1st course	P	Single-donor group (n=17) vs. multidonor group (n=27), 2nd course	P
ABC scores	53.29 ± 16.90 vs.54 ± 22.69	0.049	51.94 ± 20.40 vs.52.33 ± 19.48	0.37
CARS scores	33.64 ± 3.15 vs.34.05 ± 4.95	0.425	33.59 ± 3.06 vs.33.93 ± 4.87	0.204
SDSC scores	47.76 ± 10.24 vs.48.59 ± 16.35	0.019	45.47 ± 13.27 vs.46.52 ± 12.37	0.85

### 3.3 Assessment of adverse clinical reactions in patients who received WMT

WMT did not result in severe adverse reactions among ASD patients. Adverse reactions occurred within 24 hours, including 5 cases of diarrhea after the 1st course, which was alleviated after oral administration of antidiarrheal drugs; 4 cases of fever that reached 38.5°C, followed by a return to normal body temperature after physical cooling without recurrence; and 1 case of abdominal distension and pain around the periumbilical region, which was relieved with oral antispasmodic drug administration.

### 3.4 Compositional analysis of the gut flora before and after WMT

Stool samples from patients (n=17) at two time points, before the 1st course and after the 2nd course of WMT, were subjected to 16S rDNA sequencing. We regrouped these 17 patients as follows: (1) Patients with significantly reduced ABC and CARS scores were classified into the effective group (n = 11, 64.71%), and those with no significant change were classified into the ineffective group (n = 6, 35.29%). (2) Patients were grouped according to the donor types in the two courses, namely, the single-donor group (n = 8, 47.06%) and the multidonor group (n = 9, 52.94%).

#### 3.4.1 Changes in the alpha-diversity of the faecal flora after WMT

**Figure 1:**
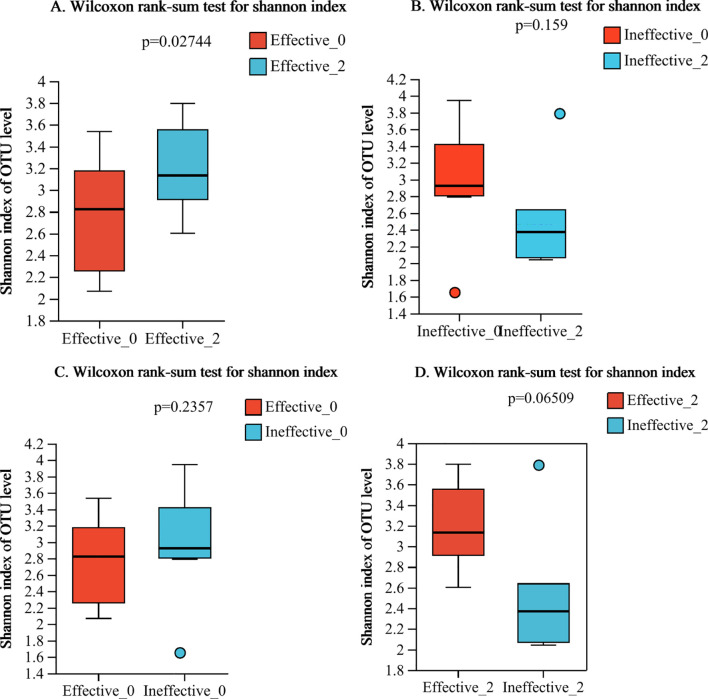
**(A–D)** Alpha diversity analysis of the effective group and ineffective group before and after WMT. Effective-0, effective group before the 1st course of WMT treatment; Effective-2, effective group after the 2nd course of treatment; Ineffective-0, ineffective group before the 1st course of WMT treatment; Ineffective-2, ineffective group after the 2nd course of treatment.

**Figure 2:**
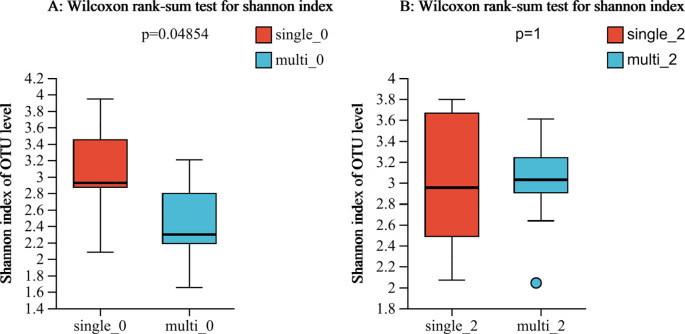
**(A, B)** Alpha diversity analysis of the single-donor group and the multidonor group before and after WMT. single-0, single-donor group before the 1st course of WMT treatment; single-2, single-donor group after the 2nd course of treatment; multi0, multidonor group before the 1st course of WMT treatment; multi2, multidonor group after the 2nd course of treatment.

In this study, we found that the alpha-diversity of the effective group increased significantly after 2 courses of treatment (Shannon index P=0.02744) (**Figure 1A**). However, that of the ineffective group slightly decreased, but the difference was not statistically significant (P>0.05) (**Figure 1B**). Compared with that at baseline, the alpha-diversity of the effective group increased after 2 courses of WMT, and that of the ineffective group decreased; however, the difference was not statistically significant (**Figures 1C, D**). Prior to the 1st course of WMT, the alpha-diversity of the single-donor group was significantly greater than that of the multidonor group, and after the 2nd course of treatment, the alpha-diversity of the multidonor group was essentially consistent with that of the single-donor group (**Figures 2A, B**).

#### 3.4.2 Changes in the composition of faecal flora after the 2nd course of WMT

**Figure 3:**
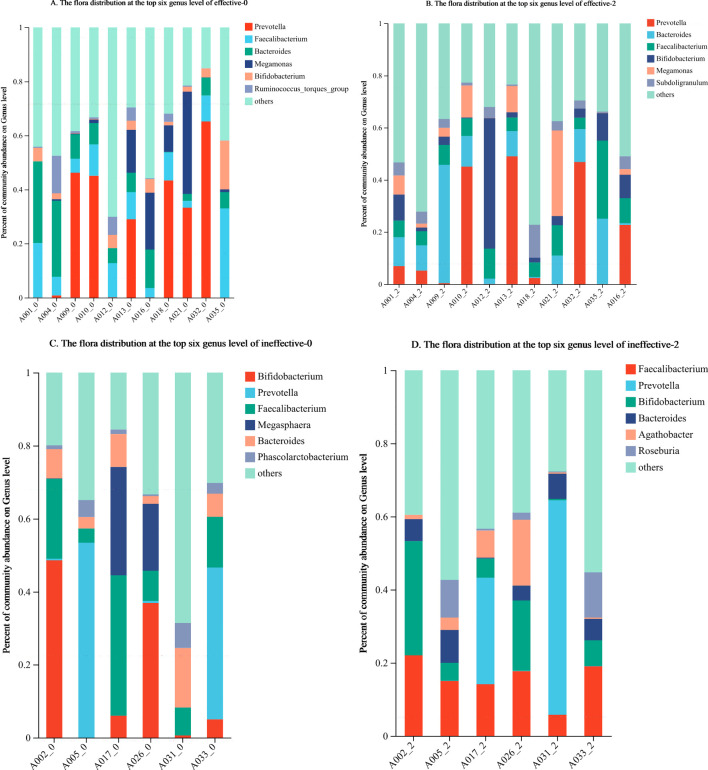
**(A–D)** Changes in the composition of the faecal flora in the effective group and ineffective group before and after WMT. Effective-0, effective group before the 1st course of WMT treatment; Effective-2, effective group after the 2nd course of treatment; Ineffective-0, ineffective group before the 1st course of WMT treatment; ineffective-2, ineffective group after the 2nd course of treatment.

At the genus level, the top 6 groups of bacteria in the effective group prior to treatment were *Prevotella, Faecalibacterium, Bacteroides, Megamonas*, *Bifidobacterium*, and *Ruminococcus_*torques_group. The top genera after the 2nd course of WMT were *Prevotella, Bacteroides, Faecalibacterium*, *Bifidobacterium*, *Megamonas*, and *Subdoligranulum*. The 5 most abundant taxa remained, and the 6th, *Ruminococcus*_torques_group, was replaced by *Subdoligranulum* (**Figures 3A, B**).

Before treatment, the top 6 groups of bacteria in the ineffective group were *Bifidobacterium*, *Prevotella, Faecalibacterium, Bacteroides, Megasphaera*, and *Phascolarctobacterium*. After the 2nd course of WMT, those in the ineffective group were *Faecalibacterium, Prevotella, Bifidobacterium, Bacteroides, Agathobacter*, and *Roseburia*. The top 4 taxa were unchanged, whereas the 5th and 6th most abundant taxa, namely, *Megasphaera* and *Phascolarctobacterium*, were replaced by *Agathobacter* and *Roseburia* (**Figures 3C, D**).

#### 3.4.3 Changes in the composition of the faecal flora in the effective group and ineffective group during the 2nd course of WMT

**Figure 4:**
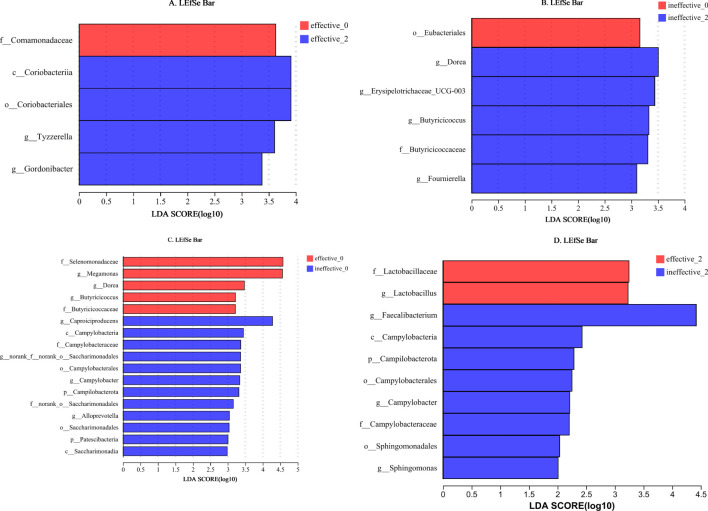
**(A–D)** LEfSe analysis of the faecal flora in the effective group and ineffective group before and after WMT. Effective-0, effective group before the 1st course of WMT treatment; Effective-2, effective group after the 2nd course of treatment; Ineffective-0, ineffective group before the 1st course of WMT treatment; Ineffective-2, ineffective group after the 2nd course of treatment.

LEfSe analysis (LDA threshold of 2) of the effective group after treatment revealed the following significant differences in the flora: (1) after treatment: c_Coriobacterlia, o_Coriobacteriales, g_Tyzzerella, and g_Gordonibacter; and (2) before treatment: f_Comamonadaceae. The following differences were detected in the ineffective group: (1) after treatment: g_Dorea, g_Erysipelotrichaceae_UCG-003, g_Butyricicoocus, f_Butyricicoccaceae, and g_Foumierella; and (2) before treatment: o_Eubacteriales (**Figures 4A, B**).

Before WMT, the significant taxa in the effective group were f_Selenomonadaceae, g_Megamonas, g_Dorea, g_Butyricicoccus, and f_Butyricicoccaceae, and the significant taxa in the ineffective group were g_Caproiciproducens, c_Campylobacteria, f_Campylobacteraceae, g_norank_f_norank_o_Saccharimonadales, o_Campylobacterales, g_Campylobacter, p_Campilobacterota, f_ nor-ank_o_Saccharimonadales, g_Alloprevotella, o_Saccharimonadales, p_Patescibacteria, and c_Saccharimonadia. After WMT, the significant taxa in the effective group were f_Lactobacillaceae and g_Lactobacillus, and the significant taxa in the ineffective group were g_Faecalibacterium, c_Campylobacteria, p_Campilobacterota, o_Campylobacterales, g_Campylobacter, f-Campylobacteraceae, o_Sphingomonadales, and g_Sphingomonas (**Figures 4C, D**).

## 4 Discussion

To date, several clinical studies have confirmed that FMT/WMT has clinical efficacy for patients with ASD, but most of these studies have been short-term studies (Kang et al., 2017; Li et al., 2021; Pan et al., 2022).Patients with ASD in this study were treated with the WMT technique, which involves the use of a fresh bacterial mixture injected through a colon TET into the patient’s intestine for 6 consecutive days for each course of treatment and a second course of treatment after 1 month. Compared with those before treatment, the clinical symptoms of the ASD patients were significantly improved at 4 and 8 weeks. Furthermore, the ABC and CARS scores were significantly lower than those at baseline, which indicated that the symptoms of ASD were alleviated after WMT and that WMT was effective for the alleviation of ASD patients’ clinical symptoms. This finding is consistent with published reports regarding the use of FMT for the treatment of ASD. After two courses of WMT, the relatedness skills and language ability of ASD patients improved significantly, as indicated by subitems of the ABC scale, and relationships with inanimate objects, adaptation to environmental changes, close feelings, and nonverbal communication also improved significantly, as indicated by subitems of the CARS. Therefore, WMT may be comprehensively beneficial in many ways for ASD patients, instead of providing certain limited improvements. However, the long-term curative effect needs to be confirmed by further follow-up observation.

One of the reasons why WMT is effective in treating diseases is that it can increase the diversity of the intestinal flora in patients. However, there is no convincing researching showing whether the selection of multiple donors or a single “super donor” is a more effective approach. However, strategies to increase the diversity of the microbiota warrant further exploration. In a randomized, double-blind, controlled study published in 2021 (Wilson et al., 2021), 87 obese adolescents were randomized into two groups and received multidonor FMT capsules (containing faecal microbiota from four lean donors of the same sex) or placebo. After six months of tracking, FMT was found to continuously changed the structure and function of the intestinal flora in the recipients; two donor microflora exhibited advantages in terms of strain implantation, suggesting the existence of a superdonor in this cohort, with high diversity and a high *Prevotella/Bacteroides* ratio. Large differences in strain implantation among recipients were found, indicating that recipient factors may also influence the effect of FMT.

At our center, obtaining fresh stool samples from a single donor for the same patient for six consecutive days is difficult. In clinical practice, receiving bacterial solutions from stool samples from different donors for 6 consecutive days during a course of WMT is relatively easy. Whether this treatment method would affect curative efficacy is unclear. Compared with that observed after single-donor transplantation, would gut flora diversity increase after receiving a multidonor transplant? Would the efficacy increase, decrease or remain unaffected? These questions have not been answered. A comparison of the ABC, CARS and SDSC scores of 44 patients with autism before and after two courses of WMT revealed that the mean scores of the three scales after treatment were significantly lower than the baseline scores. The ABC, CARS or SDSC scores did not differ between the single-donor group and the multidonor group at baseline before treatment. After the first course of treatment, the mean values of the ABC (p=0.049) and CARS scores (P=0.019) significantly differed between the single-donor group and the multidonor group, but the difference disappeared after the end of the 2nd course of treatment. In clinical practice, it is difficult to have one donor continuously provide faecal microbiota for a certain patient for multiple courses. Therefore, multidonor WMT can achieve an effect equivalent to that of an optimized single donor, which is more consistent with the actual clinical situation.

The colonization ability of the donor flora in the recipient gut after WMT may determine whether the diversity of the recipient intestinal flora can be increased, and the colonization ability of the donor flora may be related to efficacy. In a study conducted in 2021, 12 patients with ulcerative colitis were enrolled, and the dynamic changes in their faecal bacterial composition were evaluated at 4 time points: baseline prior to treatment and at 4, 12, and 18 weeks after microbiota transplantation. The results revealed that the dynamics of competition between donor and patient strains differed and that the ability to maintain bacterial colonization in inpatients varied widely over time. In one patient, significant losses in the donor flora were noted 10 weeks after faecal transplantation, which was accompanied by an increase in the number of pathogenic bacteria and a worsening of the patient’s clinical symptoms (Nanjing consensus on methodology of washed microbiota transplantation, 2020). In our study, 8 weeks after WMT, the diversity of the intestinal flora in the effective group increased significantly compared with that at baseline, whereas no increase was identified in the ineffective group. These findings suggest that the flora could not colonize the gut of patients in the ineffective groups long after receiving the donor intestinal flora, which led to poor clinical efficacy. This lack of colonization may be related to the donor flora, compatibility of the recipient flora, immune interactions, etc. Increasing the number of treatment courses and changing donors may be one of the solutions, but more research is needed to verify this.

The alpha-diversity of the single-donor group before WMT treatment was significantly greater than that of the multidonor group, and the diversity of the multidonor group after WMT treatment was essentially consistent with that of the single-donor group (**Figure 2**), which indicates that multidonor treatment can significantly improve the intestinal flora diversity of patients. Moreover, the microbiota diversity in the single-donor group remained essentially unchanged after treatment. After treatment, the alpha-diversity did not significantly differ between the two groups, which is consistent with the lack of significant difference in clinical improvement between the two groups. The number of patients in this study was small, which may limit the interpretability of the findings.

The specific microorganisms that play a main role in the treatment of ASD patients with WMT remain unknown. Whether certain microbial communities are targeted remains to be further investigated. In our study, we attempted to identify the microbial communities related to treatment outcomes. Faecal sequencing of healthy children and children with ASD revealed that at the genus level, the dominant taxa in the feces of children with ASD were *Megamonas, Megasphaera*, *Barnesiella, Pseudomonas*, etc (Zhao et al., 2019). In this study, prior to WMT, the ineffective group and the effective group were similar in terms of the top 6 dominant intestinal bacterial taxa, which included *Prevotella, Faecalibacterium, Bacteroides*, and *Bifidobacterium*. The difference was that the effective group was richer in *Megamonas* and the *Ruminococcus*_torques_group prior to treatment, whereas the ineffective group was richer in *Megasphaera* and *Phascolarctobacterium*. This finding suggests a clear difference in the predominant intestinal biota prior to treatment between the two groups of patients with ASD. The enrichment of *Megamonas* and the *Ruminococcus*_torques_group in the gut prior to treatment was associated with better efficacy. In contrast, the enrichment of *Megasphaera* and *Phascolarctobacterium* may be related to their lower effectiveness. Therefore, we can sequence and analyze the intestinal flora of patients with ASD prior to WMT, and treatment may be more effective when *Megamonas* and the *Ruminococcus*_torques_group are more abundant. When *Megasphaera* and *Phascolarctobacterium* are more abundant, transplantation may have negligible effects.

The exact relationship between the gut flora and autism is currently unknown, and different studies have reached different conclusions (Liu et al., 2019). *Lactobacillus* is more abundant in people with autism than in healthy people (Tomova et al., 2015; Strati et al., 2017). However, consensus regarding differences in the abundance of *Faecalibacterium* between the gut of people with ASD and that of healthy individuals is lacking, with both an increase and a decrease reported (Williams et al., 2011; De Angelis et al., 2015; Inoue et al., 2016). In this study, a comparison of the effective group and the ineffective group after WMT revealed that *Lactobacillus* was significantly enriched in the effective group, whereas *Faecalibacterium*, *Campylobacter*, and *Sphingomonas* were significantly enriched in the ineffective group. The increase in *Lactobacillus* abundance in the effective group may have been related to improvements in efficacy. The increase in *Faecalibacterium*, *Campylobacter*, and *Sphingomonas* abundances in the ineffective group may be related to poor treatment efficacy.

## 5 Limitations

The aetiology of ASD remains unclear, and drug treatments that can significantly improve the condition are currently lacking. Existing drug and nondrug therapies do not meet expectations in terms of their ability to improve the core symptoms of ASD. WMT is expected to become a relatively effective treatment approach to improve the core symptoms of ASD. This study provides sufficient clinical evidence for the short-term efficacy of WMT in the treatment of ASD and preliminarily explores the changes in the microbiota after treatment; however, the following limitations remain:

1. Forty-four patients were included in this study, including a single-donor treatment group (17 patients) and a multidonor treatment group (27 patients). The sample size was small, and in follow-up studies, the sample size of single- and multidonor WMT groups should be increased to further analyze differences in clinical efficacy.
2. The follow-up time was short, and the ability of WMT to improve the long-term prognosis of patients with ASD is unclear. In the future, more prospective, large-sample-size and long-term follow-up studies are needed to further confirm our findings and conduct in-depth research.
3. To date, a variety of mechanisms related to the function of the gut microbiota in ASD have been proposed. This study provides an explanation for the changes in the gut microbiota after WMT, and basic experiments can be conducted to explore the specific pathways underlying the mechanism of action of the gut microbiota. The development of WMT-based therapies that target the microbiota related to ASD may be a specific treatment direction for ASD.

## 6 Conclusions

1. After two courses of WMT, the ABC, CARS and SDSC scores of ASD patients decreased compared with those before treatment, indicating that the core symptoms and sleep disorders of patients with autism can improve rapidly after WMT and that the short-term efficacy of WMT is good.
2. The improvements in the ABC, CARS and SDSC scores did not obviously differ between the single- and multidonor groups after two courses of WMT. This finding suggests that the use of multiple donors or a single donor may have the same efficacy.
3. WMT is safe and causes few adverse reactions in the treatment of children with autism.
4. The increase in faecal microbiota alpha-diversity and changes in the abundance of some microbial taxa may be related to the efficacy of WMT.

## Data Availability

Raw sequencing data have been deposited in the NCBI Sequence Read Archive database under study accession number PRJNA1282650.
